# Disease Severity- and Hormonal Status-Dependent Alterations of EGF and MIF in the Serum of Endometriosis Patients

**DOI:** 10.3390/ijms26146695

**Published:** 2025-07-12

**Authors:** Norbert Tóth, Réka Brubel, Attila Bokor, Ágnes Kemény, Nelli Farkas, Tibor Pál, Zsuzsanna Helyes, Krisztina Pohóczky

**Affiliations:** 1 Department of Pharmacology and Pharmacotherapy, Medical School, University of Pécs, 7624 Pécs, Hungary; toth.norbert@pte.hu (N.T.); kemenyagnes1@gmail.com (Á.K.); pal.tibor@outlook.com (T.P.); pohoczky.krisztina@pte.hu (K.P.); 2 1st Department of Obstetrics and Gynecology, Semmelweis University, 1082 Budapest, Hungary; brubelreka@gmail.com (R.B.); attila.z.bokor@gmail.com (A.B.); 3 Department of Physiology and Biochemistry, University of Veterinary Medicine, 1078 Budapest, Hungary; 4 Department of Medical Biology and Central Electron Microscope, Medical School, University of Pécs, 7624 Pécs, Hungary; 5 Institute of Bioanalysis, Medical School, University of Pécs, 7624 Pécs, Hungary; nelli.farkas@aok.pte.hu; 6 Hungarian Research Network, Chronic Pain Research Group (HUN-REN-PTE), 7624 Pécs, Hungary; 7 PharmInVivo Ltd., Szondi György Str. 10, 7629 Pécs, Hungary; 8 Department of Pharmacology, Faculty of Pharmacy, University of Pécs, 7624 Pécs, Hungary

**Keywords:** endometriosis, systemic inflammation, interleukins, somatostatin, tumor necrosis factor alpha, macrophage migration inhibitory factor, calcitonin gene-related peptide, visual analogue scale, pain assessment, immunoassay

## Abstract

Endometriosis is the extrauterine engraftment of endometrium-like tissue, causing chronic pain. Complex sensory–vascular–immune interactions, including growth factors, cytokines, and neuropeptides, are implicated in its pathophysiology, but the mechanisms remain unknown. Here, epidermal growth factor (EGF), vascular endothelial growth factor, interleukins (IL-1β, IL-6, IL-8), macrophage migration inhibitory factor (MIF), calcitonin gene-related peptide, and somatostatin were measured in the serum of endometriosis patients with different disease severities, menstruation cycle- and pharmacotherapy-related hormonal status compared with controls. Mediator levels in deep-infiltrating rectosigmoid nodules were also compared with those in non-endometriotic colon tissues. Pain was assessed by the visual analogue scale. Serum EGF was significantly lower in mild endometriosis and in the secretory phase. MIF and IL-6 were higher in stage I–IV endometriosis, with MIF also higher in the secretory phase and in patients not receiving oral contraceptives. Somatostatin was lower in mild endometriosis than that in healthy individuals and the severe endometriosis group. No tissue-level differences were found. A strong positive correlation between serum EGF and somatostatin levels and dysmenorrhea and dysuria was detected in mild cases. It is concluded that certain serum alterations may be related to severity- and hormone status-dependent endometriosis mechanisms, but their diagnostic/prognostic value seems to be limited due to variability and lack of specificity.

## 1. Introduction

Endometriosis is a complex estrogen-dependent inflammatory disease that affects approximately 10–15% of women of premenarchal, reproductive, and postmenopausal ages [1,2]. The initial diagnosis of endometriosis relies on a detailed medical history, thorough gynecological examination, and diagnostic tests including imaging techniques, laparoscopy, and biochemical or pathological analyses. Despite advances in diagnostic techniques, patients often face suboptimal efficacy under current treatments with adverse side effects and a high recurrence rate [3,4]. The severity of endometriosis is typically assessed with the help of classification systems such as the revised American Society for Reproductive Medicine (rASRM) score and the ENZIAN system, which was introduced in 2020/2021 [5,6]. The ENZIAN system evaluates lesion size, localization, and depth of infiltration. 

The primary symptoms of endometriosis include painful menstrual bleeding (dysmenorrhea, DM) [7], painful sexual intercourse (dyspareunia) [8], painful defecation (dyschezia) [9], painful urination (dysuria) [10], severe chronic pelvic pain (CPP) [11], and infertility. Endometriosis-associated CPP (endo-CPP) is characterized by multiple pain mechanisms, including nociceptive, neuropathic, and nociplastic components. In clinical practice, the intensity of these pain symptoms is commonly assessed using the visual analogue scale (VAS), a well-established and reliable tool that rates endometriosis-related pain on a scale from 0 (no pain) to 10 (worst imaginable pain) [12,13,14]. Importantly, several studies [15,16] have reported that the severity or anatomical localization of lesions does not necessarily correlate with pain intensity, but the exact biological explanation remains poorly understood. Moreover, although endometriosis has historically been considered a pelvic disorder, it is increasingly recognized as a systemic disease involving immune, vascular, and neural components [17,18,19,20].

Recent studies have highlighted the significance of immune-, vascular-, and neuro interactions in the pathogenesis of endometriosis-associated pain, suggesting a complex crosstalk between peripheral nerves, immune cells, and inflammatory mediators, leading to the sensitization of nociceptive pathways and contributing to nociplastic and neuropathic pain components. Key mediators involved in this complex mechanism include pro-inflammatory cytokines such as interleukins (IL)-6, -8, and -1β; tumor necrosis factor α (TNF-α); macrophage migration inhibitory factor (MIF); growth factors including epidermal growth factor (EGF) and vascular endothelial growth factor (VEGF); and neuropeptides such as calcitonin gene-related peptide (CGRP) and somatostatin (SOM) [21,22,23].

IL-6 is a pro-inflammatory cytokine that has been found to be elevated in the serum and peritoneal fluid of women with endometriosis, suggesting a potential role in the disease’s pathology [24]. IL-8 is a potent pro-inflammatory cytokine showing increased levels in the peritoneal fluid and lesions of endometriosis patients. It has been shown to stimulate endometrial cell proliferation and to contribute to disease progression [25,26]. IL-1 generally regulates inflammation and, together with TNF-α and IL-6, has been found to promote multiple aspects of endometriosis, including inflammation, nerve growth factor expression, angiogenesis, and the growth and adhesion of ectopic endometrial tissue [27,28]. EGF, involved in tissue remodeling and cellular proliferation, is dysregulated in endometriosis and has been associated with cell migration and invasion [29]. VEGF has been detected in both peritoneal fluid and plasma samples of patients with endometriosis; however, data regarding its protein levels remain contradictory [30,31]. MIF is a pro-inflammatory cytokine that promotes inflammation and is found in higher concentrations in women with advanced-stage endometriosis, suggesting its involvement in disease progression [32]. CGRP, a neuropeptide implicated in neurogenic inflammation and tissue damage, has recently been associated with endometriosis [33]. SOM is a well-characterized anti-nociceptive and anti-inflammatory neuropeptide that may play a role in pain modulation; however, its involvement in endometriosis is poorly understood [23].

Although the individual roles of several of these molecules have been investigated in previous endometriosis-related studies, data on their associations with disease severity and hormonal status are limited. Therefore, we aimed to investigate their serum concentrations in relation to the disease severity, menstrual cycle phase, and hormonal treatment in well-characterized patient cohorts. In addition, the tissue levels of these mediators were analyzed in severe, deep-infiltrating rectosigmoid (DIER) lesion homogenates and correlated with patient-reported pain intensities.

## 2. Results

### 2.1. Patient Demographics and Pain Parameters of Study Participants

The menstrual phase, parity, medication, and VAS pain scores of endometriosis patients who provided serum samples, as well as the menstrual phase, parity, and medication of control subjects, are summarized in Table 1.

The endometriosis patients were similar in terms of menstrual phase; however, the mean age of the patients with endometriosis was significantly higher than that of the healthy controls. The severity of pain symptoms—based on VAS scores—did not differ significantly between patients with mild or severe endometriosis. There were no significant differences in age or menstrual cycle length between patients with DIER endometriosis patients and control subjects who provided tissue samples.

### 2.2. Serum Cytokine and Neuropeptide Alterations in Endometriosis

**Figure 1:**
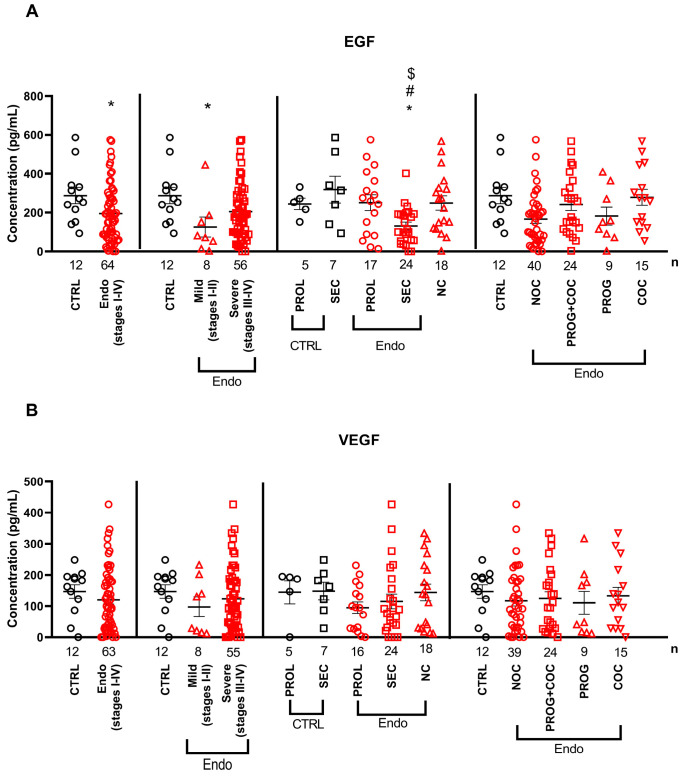
Serum levels of (**A**) epidermal growth factor (EGF) and (**B**) vascular endothelial growth factor (VEGF) in endometriosis patients compared with healthy individuals. Horizontal lines representing the means ± SEM are demonstrated together with individual data points in a scatter plot. Abbreviations: COCs: combined oral contraceptives; NC: no cycle, NOCs: no oral contraceptives; PROG: progesterone; PROL: proliferative phase of menstrual cycle; SEC: secretory phase of menstrual cycle. *p* < 0.05; * vs. CTRL group (respective cycle if applicable): # vs. proliferative cycle of respective group; $ vs. NC group (Mann–Whitney U-test (CTRL vs. Endo), Kruskal–Wallis + Benjamini–Hochberg post hoc test (rest of the comparisons)).

The serum EGF levels were significantly lower in patients with endometriosis (stages I–IV) than those in healthy controls, with the most pronounced decrease observed in the mild stages of the disease. In addition, EGF levels were lower in the secretory phase of endometriosis patients (ENDO SEC group) than in the secretory phase of healthy individuals (CTRL SEC group), in the proliferative phase (ENDO PROL group), and in endometriosis patients without a menstrual cycle (NC group). This growth factor was not influenced by any hormonal treatments (Figure 1A). The serum VEGF levels remained unchanged in all comparisons (Figure 1B).

The serum MIF concentrations were higher in patients with endometriosis (stages I–IV) than those in healthy controls, even when mild and severe subgroups were analyzed separately. Within the endometriosis group, it was higher in the secretory phase (ENDO SEC) than in proliferative (ENDO PROL) and NC patients. Furthermore, serum MIF levels were higher in patients not receiving hormonal therapy (ENDO NOC) than in controls.

**Figure 2:**
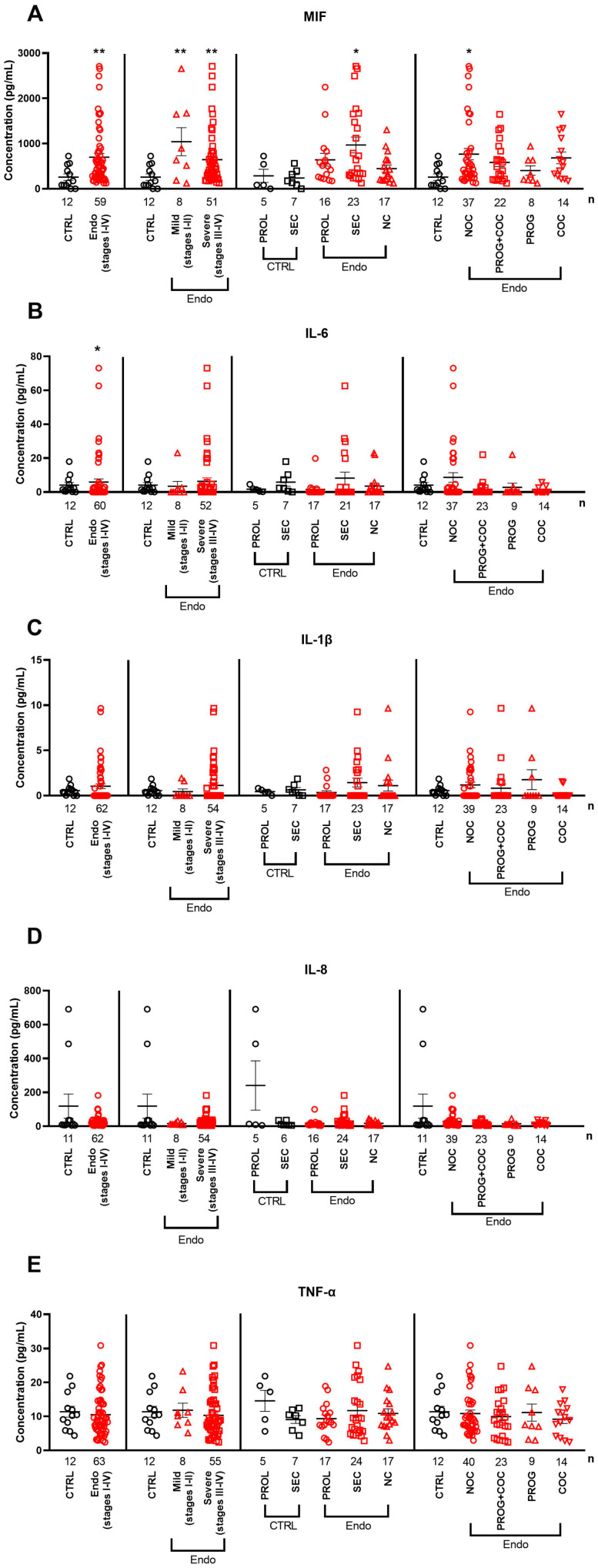
Serum levels of (**A**) macrophage migration inhibitory factor, (**B**) interleukin 6, (**C**) interleukin-1β, (**D**) interleukin 8, and (**E**) tumor necrosis factor α in multiple comparisons between control and endometriosis samples. Horizontal lines representing the means ± SEM are demonstrated together with individual data points in a scatter plot. Abbreviations: COCs: combined oral contraceptives; NC: no cycle, NOCs: no oral contraceptives; PROG: progesterone; PROL: proliferative phase of menstrual cycle; SEC: secretory phase of menstrual cycle. **: *p* < 0.01; * *p* < 0.05; Mann–Whitney U-test (CTRL vs. Endo), Kruskal–Wallis + Benjamini–Hochberg post hoc test (rest of the comparisons).

IL-6 was significantly higher in the stage I–IV endometriosis group than that in healthy controls, but its concentration was not affected by disease severity, menstrual cycle phase, or medication status. None of the other investigated interleukins (IL-1β and IL-8) and TNF-α showed any differences in the serum of endometriosis patients in any comparison (Figure 2C–E).

**Figure 3:**
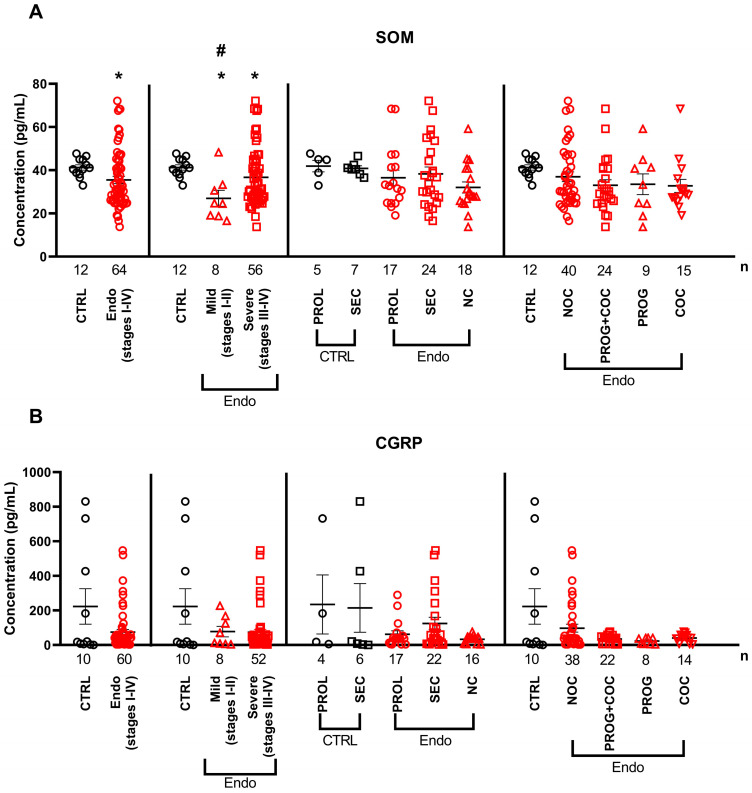
Serum levels of (**A**) somatostatin and (**B**) calcitonin gene-related peptide in multiple comparisons between control and endometriosis samples. Horizontal lines representing the means ± SEM are demonstrated together with individual data points in a scatter plot. Abbreviations: COCs: combined oral contraceptives; NC: no cycle, NOCs: no oral contraceptives; PROG: progesterone; PROL: proliferative phase of menstrual cycle; SEC: secretory phase of menstrual cycle. *p* < 0.05; *: vs. ctrl; # vs. mild endometriosis group; Mann–Whitney U-test (CTRL vs. Endo), Kruskal–Wallis + Benjamini–Hochberg post hoc test (rest of the comparisons).

SOM levels were significantly lower in the serum of endometriosis patients than those in controls, both when all the stages were analyzed together (stages I–IV) and when mild and severe stages were analyzed separately. In addition, significantly lower concentrations were observed in mild cases than those in severe endometriosis (Figure 3A). Regarding serum neuropeptide levels, CGRP showed no significant difference between healthy subjects and endometriosis patients in all the comparisons (Figure 3B).

### 2.3. Growth Factors and Cytokines Are Not Elevated Locally in Severe Endometriosis Lesions

**Figure 4:**
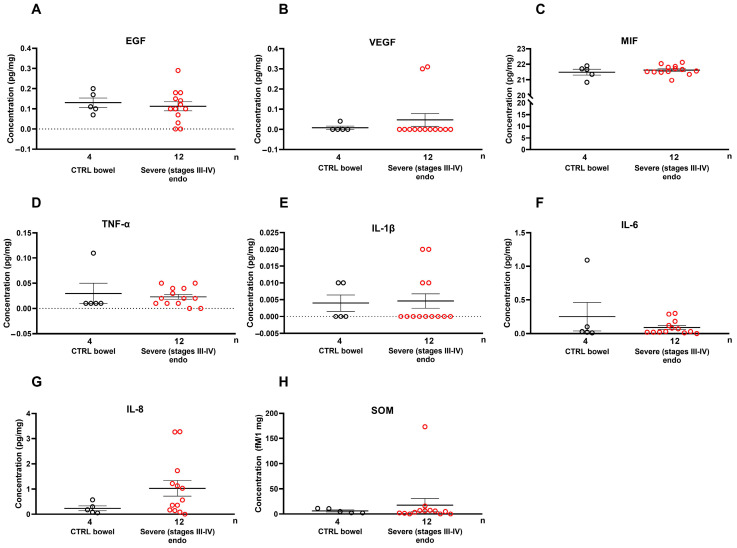
Tissue levels of (**A**) epidermal growth factor, (**B**) vascular endothelial growth factor, (**C**) macrophage migration inhibitory factor, (**D**) tumor necrosis factor α, (**E**) interleukin-1β, (**F**) interleukin 6, (**G**) interleukin-8, and (**H**) somatostatin in comparisons between healthy bowel wall (*n* = 4) and deep-infiltrating rectosigmoid endometriosis (DIER; *n* = 12) samples. Horizontal lines representing the means ± SEM are demonstrated together with individual data points in a scatter plot; Mann–Whitney U-test.

In endometriosis tissue homogenates, we did not observe any changes in growth factors or cytokines between the implanted endometriosis lesion and the normal intestinal wall, suggesting that the endometriosis lesion itself does not secrete these factors (Figure 4).

### 2.4. Serum EGF and SOM Showed Positive Correlations with Pain Parameters in Mild Endometriosis

In patients with mild endometriosis, EGF showed a strong positive correlation with dysuria (r = 0.8; *p* = 0.048). Similarly, SOM also showed a strong positive correlation with dysuria in this subgroup (r = 0.8; *p* = 0.048). However, when all the stages of endometriosis (I–IV) were analyzed collectively, the analyses did not reveal any significant correlations between serum or tissue levels of the measured molecules and individual pain scores or the composite pain score. The results of the correlation analyses are presented in Supplementary Figures S2–S12.

## 3. Discussion

We present the first data on disease severity-associated changes in the serum levels of the growth factor EGF, the inflammatory cytokines MIF and IL-6, and the inhibitory sensory neuropeptide SOM in patients with endometriosis. Among these, EGF and MIF also showed menstrual cycle-dependent alterations, while IL-6 and SOM did not exhibit hormonally driven changes. Furthermore, EGF and SOM levels were significantly corelated with DM and dysuria in patients with mild endometriosis, suggesting a potential link between these mediators and pain symptoms independent of hormonal status.

Early and non-invasive diagnosis of endometriosis is critically important, and considerable efforts have been made to identify diagnostic and prognostic biomarkers. Nowadays, advanced imaging techniques such as high-resolution MRI and transvaginal ultrasound have become increasingly reliable for detecting ovarian and deep-infiltrating endometriotic lesions. However, the systemic and tissue-specific levels of key mediators involved in inflammation and endometriosis-related pain—as well as their modulation by menstrual cycle and hormonal treatments—still require further investigations. Moreover, the existing literature presents numerous, yet often contradictory, findings regarding the roles of various growth factors, chemokines, and cytokines, further complicating the interpretation of their contribution to the development or progression of endometriosis [34].

EGF has been implicated in the pathogenesis of endometriosis due to its well-known roles in cell proliferation, differentiation, and migration; however, limited data are available regarding its systemic and tissue-specific levels in this disease [29,35,36,37]. We observed a significant decrease in systemic EGF levels when evaluating all the patients together (stages I–IV) and specifically in the minimal–mild stages (I–II). Additionally, its concentration was influenced by the menstrual cycle, with lower levels observed in the secretory phase under gestagen dominance, while no difference was measured in tissue samples. Similar results have been found in certain malignancies, including thyroid cancer, non-small-cell lung cancer, and head and neck carcinomas. Moreover, endometriosis cell lines with low EGF secretion show more dedifferentiated rather than proliferative properties [38,39,40]. We found that EGF showed a strong positive correlation with DM and dysuria in patients with mild endometriosis, suggesting that serum EGF levels are associated with reduced pain symptoms in mild disease. In the literature, EGFR activation has been associated with cancer-related neuropathic pain; therefore, EGFR inhibition may represent a potential therapeutic approach for certain types of pain [36,41,42]. However, further studies are necessary to elucidate the precise roles of EGF and EGFR in endometriosis and related pain.

Increased serum, but not tissue, MIF concentrations were detected in stages I–IV in patients with mild and severe endometriosis, with the highest levels observed in stages III and IV, as well as in patients not receiving oral contraceptives. Elevated serum and tissue MIF levels have been reported in response to estrogen in both rat and human endometrial tissue and endometriosis lesions [32,43,44], which strongly supports our findings. Additionally, significantly higher serum MIF levels were found in stage III-IV patients with active lesions but not in those with chronic fibrotic conditions associated with infertility and pelvic pain [45,46,47]. Based on these data, hormonal treatments may exert beneficial effects on macrophage infiltration and inflammation by reducing MIF levels.

Most studies have examined IL levels in endometriosis patients to identify potential biomarkers but have reported highly contradictory results [34,48,49]. In our study, only IL-6 showed significant elevation in the serum of endometriosis patients independently of disease severity, menstrual cycle, or medication and did not change in the tissue. It has been reported that IL-6 levels in the peritoneal fluid of patients with minimal–mild endometriosis do not correlate with pain; however, this relationship has not been investigated in serum or tissues obtained from patients with DIER [50]. IL-8 has been found to be elevated in various pathologies causing CPP associated with endometriosis [26,51,52,53]. IL1-β, together with TNF-α, has been shown to stimulate IL-8 production, contributing to the inflammatory milieu and pain [54,55]. Based on our data and the existing literature, IL-1β, IL-6, and IL-8 do not appear to be reliable predictive serum biomarkers for endometriosis, as they show high interindividual variability.

SOM is a neuropeptide that inhibits the release of various hormones and neurotransmitters. In this study, decreased SOM levels were observed in the serum of endometriosis patients compared with the control group, including the mild subgroup when analyzed separately. Although the literature reported lower SOM mRNA in mild and severe endometriosis tissue samples, we did not find altered SOM protein levels in severe DIER lesions compared with normal bowel [56]. Therefore, most of the SOM measured in the systemic circulation is likely to be of a sensory neural origin and is not derived from endometriosis lesions. Furthermore, since the samples were harvested after 12 h of fasting, the release of SOM from the gastrointestinal tract can be excluded [57]. The sensory neural release of SOM into the systemic circulation is also supported by a wide range of our previous studies [58,59]. Endometriosis lesions are typically densely innervated by peptidergic sensory fibers, which release SOM in response to inflammatory mediators. SOM has been shown to inhibit cancer cell proliferation and invasion, inflammation, pain, and VEGF-induced neovascularization [23,60,61,62,63]. Therefore, the sustained release of SOM from sensory fibers may serve as an endogenous defense mechanism [64]. This counter-regulatory mechanism may operate during the early stages of endometriosis; however, the low serum SOM levels measured in our study may reflect depletion caused by long-term stimulation. Additionally, SOM levels showed a strong positive correlation with dysuria in patients with stage I–II endometriosis. SOM is known to have effects on pain pathways by inhibiting nociceptive and inflammatory processes, potentially through SSTR4 pathways [65]. Studies have also shown that a decrease in SOM levels can lead to increased pain sensitivity [66,67].

Angiogenic factors play a crucial role in the development of both malignant and benign pathologies, including diabetic retinopathy, ischemic diseases, autoimmune disorders, and connective tissue diseases. In endometriosis these factors promote the vascularization of endometriotic lesions, facilitating the invasion of ectopic cells and the transport of inflammatory mediators [68,69,70]. Therefore, VEGF has also been investigated as a biomarker in endometriosis [30]. In our patient cohort, both serum and tissue VEGF levels remained unchanged compared with those in the control groups, suggesting that systemic or local VEGF does not contribute to the disease’s development, severity, or symptomatology. The literature data support our findings as no significant differences in serum VEGF levels were found between women with endometriosis-associated and tubal infertilities [31] or between patients with different types of endometriosis and healthy individuals [49,71].

TNF-α is one of the most common pro-inflammatory and proangiogenic factors, produced mainly by macrophages and Th1 cells. TNF-α, together with IL1-β, was shown to stimulate IL-8 production, contributing to the inflammatory milieu and pain in endometriosis [54,55]. Although several studies suggest that it may serve as a potential biomarker [72], data on TNF-α in endometriosis is highly controversial, with some studies showing elevated levels [73,74], and others finding no difference [48,49]. We also found no difference in systemic or local TNF-α levels in endometriosis patients in any comparison.

CGRP has recently been linked to endometriosis and endometriosis-associated pain, with recent publications suggesting its role in the crosstalk between the nervous and immune systems, promoting both pain signaling and lesion progression [33,75,76]. However, in our samples, no alterations in CGRP levels were observed in either serum or tissue. This does not exclude the possibility that CGRP blockers may be effective in the treatment of endometriosis, as CGRP has been shown to enhance the proliferation of endometriosis cells in vitro [33,77], but its potential as a biomarker is highly questionable.

The main limitation of our study is the relatively small size of the subgroups. However, rigorous patient selection, precise disease staging, and comprehensive subgroup analysis enhance the reliability of our results. With these methodological strengths, this study provides valuable insights into disease severity- and hormone-dependent systemic alterations of key mediators associated with inflammation and pain in endometriosis. Another limitation is the descriptive nature of these results, but it is important to emphasize that the current literature presents contradictory findings regarding several of the studied markers (e.g., EGF, MIF, IL-6, IL-8, TNFα, CGRP) in endometriosis and none of them were studied in deep-infiltrating endometriosis lesions before. These results can serve as a reliable basis for future hypothesis-driven studies.

## 4. Materials and Methods

### 4.1. Sample Collection

Blood samples of endometriosis patients were collected before surgery on the same day in the morning. The collected blood samples were allowed to coagulate at room temperature and centrifuged for 10 min at 3000 rpm to separate the cellular elements. Subsequently, the serum was decanted, aliquoted, and stored at −80 °C until the analysis. Surgical specimens were collected from 2018 to 2021 at the 1st Department of Obstetrics and Gynecology, Faculty of General Medicine, Semmelweis University, Budapest (26058-5/2020/EÜIG, license provided: 6 July 2020). Endometriotic tissue collection was carried out right after the induction of anesthesia before any surgical manipulations to avoid thermal and physical damage of the collected samples. Control bowel wall specimens were obtained from histologically normal segments of resected bowel tissue, collected at least 5 cm away from the surgical resection margin. The extracted DIER nodules were immediately frozen in liquid nitrogen and stored at −80 °C until further analysis.

For participation in the study, the inclusion criteria were age between 15 and 40 years, infertility, and/or pain or previous medical confirmation of endometriosis. The exclusion criteria were the presence of any autoimmune disease, chronic inflammatory conditions, insulin resistance, diabetes, psoriasis, irritable bowel syndrome, colitis ulcerosa, bronchial asthma, immunosuppressant treatment, mental illnesses, diagnosed or suspected malignant neoplasms, and smoking.

The clinical data of the patients were collected; informed consent forms were completed and signed; and the preoperative status was assessed in the days before the surgeries (Table 1). The stage and severity of endometriosis were determined using the revised American Society for Reproductive Medicine (rASRM) scoring system (formerly known as the revised American Fertility Society—rAFS system), which classifies the disease into stages based on the number of lesions and depth of infiltration: minimal (stage I), mild (stage II), moderate (stage III), and severe (stage IV) [6]. Additionally, the #ENZIAN classification system (endometriosis: E, the nature of endometriosis: N, and the zygosity of endometriosis: Z) was used to provide a more precise morphological description of deep-infiltrating endometriosis [5]. The ENZIAN scores of the patients who provided serum samples are listed in Supplementary Figure S1.

Control serum samples were obtained from healthy subjects. The endometriosis patients were categorized as having mild (rASRM stage I–II, *n* = 8) or severe (rASRM stage III–IV, *n* = 51–56) disease. Serum samples were further categorized into three groups based on menstrual cycle phase: a proliferative phase (PROL) (*n* = 16–17) and secretory phase (SEC) (*n* = 21–24) groups were created for both control and endometriosis patients, and the no cycle group (NC) (*n* = 16–18) only included endometriosis patients with medically suppressed cycles.

Pharmacological status was classified as follows: all the control subjects were medication-free. Within the endometriosis group, five subgroups were defined: no oral contraceptives (NOCs) (*n* = 37–40): patients not taking any hormonal treatments; progesterone (PROG) (*n* = 8–9): patients taking progesterone-only oral contraceptives; combined oral contraceptives (COCs) (*n* = 14–15): patients taking contraceptives containing both estrogen and progesterone; progesterone + COCs (*n* = 22–24): patients with overlapping treatment histories from the PROG and the COC subgroups.

### 4.2. VAS Collection

The VAS is considered one of the most widely accepted clinical tools for pain assessment. In this study, endometriosis patients rated the intensity of specific pain symptoms including DM, dyspareunia, dyschezia, dysuria, and CPP on the scale from 0 (“no pain”) to 10 (“worst imaginable pain”) prior to blood sampling and tissue collection [13]. A composite pain score was also calculated by summing the individual VAS scores for each symptom.

### 4.3. Sample Preparation

Blood samples were collected using BD Vacutainer^®^ tubes (Becton, Dickinson and Company, 1 Becton Drive Franklin Lakes, NJ, USA, 07417-1880) centrifuged at 200× *g* for 15 min followed by 3220× *g* for 15 min.

Tissue samples for the Luminex, ELISA, and RIA assays were snap-frozen, pulverized, and homogenized in ice-cold PBS containing 10 mg/mL phenylmethanesulfonylfluoride fluoride (PMSF, Sigma Aldrich, P7626, St. Louis, MO, USA) and protease inhibitor (Cat. No.: P8340, Merck Millipore, Merck KGaA, Darmstadt, Germany). Homogenates were centrifuged at 3225× *g* for 20 min at 4 °C, and the supernatants were collected.

A CGRP-free serum sample was prepared using CGRP Affinity Sorbent (Cat. No.: A19482, Bertin bioreagents, Montigny le Bretonneux, France). Briefly, 1 mL of sorbent was incubated with 20 mL control serum overnight at 4 °C under gentle agitation. The mixture was then passed through an empty column, and the CGRP-depleted serum was collected.

### 4.4. Measurement of Inflammatory Cytokine Concentration Using Luminex xMAP Technology

Serum and tissue concentrations of IL-1β, IL-6, IL-8, TNF-α, VEGF, and EGF were measured using a customized Milliplex Human Cytokine/Chemokine Magnetic Bead Panel (Merck Millipore, Merck KGaA, Darmstadt, Germany). All the samples were tested undiluted, in duplicate, and in a blinded fashion, following prior optimization and the manufacturer’s protocol.

Briefly, 25 µL of each sample, control, or standard was added to 25 µL of antibody- and a color-coded magnetic bead mixture. After overnight incubation at 4 °C, the beads were washed and incubated with biotinylated detection antibodies followed by streptavidin-PE. After the final washing steps, the beads were re-suspended in 150 µL of drive fluid and analyzed using the Luminex MagPix instrument (Merck Millipore, Merck KGaA, Darmstadt, Germany). Five-PL regression curves were used to plot the standard curves for all the analytes by the Belysa 1.1 software analyzing the bead classification and reporter median fluorescence intensities. For tissue samples, the results (pg/mL) were normalized to tissue weights. The assay variability (intra- and inter-assay coefficients) is detailed in Supplementary Table S1.

### 4.5. CGRP, MIF, and SOM Measurement with ELISA

The CGRP levels in endometriosis serum and tissue samples were determined using the Biovendor Human CGRP sandwich ELISA Kit (Cat. No.: RA19021R, Biovendor Laboratorní medicína a.s., Brno, Czech Republic) according to the manufacturer’s instructions [78]. Briefly, 100 µL of standards, quality controls, and samples were added to a 96-well plate and incubated overnight at 4 °C with gentle agitation. After washing, 200 µL of substrate solution was added, and the plate was covered and incubated in the dark at room temperature (RT). The absorbance was measured at 405 nm with a Labsystems DC plate reader (LabSystems; Texas, TX, USA). The results are expressed as pg of CGRP/mL to total protein content.

MIF concentrations were measured using the ThermoFisher Human MIF ELISA kit (Cat. No.: EHMIF; ThermoFisher; Waltham, MA, USA). The procedure followed the manufacturer’s instructions. After the initial incubation and washing steps, 100 µL of biotin-conjugated antibody and 100 µL of streptavidin-HRP were added with the respective incubations. Following TMB Substrate development and stoppage, the absorbance was measured at 450 nm. The results are expressed as pg of MIF/mL of total protein content.

SOM levels in the serum samples were quantified using the Cloud-Clone Corp. ELISA kit (Cat. No.: CEA592Hu; Cloud-Clone Corp., TX, USA) [79]. After incubation with Detection Reagent A, the plates were washed and incubated in the dark. Following further washes, the plates were incubated with Detection Reagent B, and the substrate was added to all the samples. After color development, the plates were read at 450 nm with the plate reader immediately. The results are expressed as pg of SOM/mL of total protein content of the samples. For tissue samples, the results (pg/mL) were normalized to tissue weights. The assay variability (intra- and inter-assay coefficients) is detailed in Supplementary Table S2.

### 4.6. Radioimmunoassay

The SOM levels in tissue homogenates were measured using a radioimmunoassay (RIA) with a somatostatin-14 antibody generated against synthetic somatostatin-14 peptide [58]. Standards (80–10,000 fM/mg) were prepared from the synthetic peptide, and the concentrations were calculated based on a standard calibration curve. The tissue samples were homogenized as described in Section 2.3. The supernatants were collected, transferred to cold tubes, and centrifuged at 10,000× g for 15 min at 4 °C. RIA tubes (Merck, Darmstadt, Germany) were loaded with 100 μL standard or 50 μL unknown sample, 100 μL antiserum (1: 7500 dilution), 100 μL of I^125^ isotope-labeled antigen (3000 cpm/ tube), and 750 µL of RIA buffer [0.05 mol/L (pH 7.4) phosphate buffer containing 0.1 mol/L NaCl, 0.25% (*w*/*v*) BSA (Sigma Aldrich, A7906, St. Louis, MO, USA ), and 0.05% (*w*/*v*) NaN_3_]. The samples were incubated at 4 °C for 48–72 h. For separation, 100 μL of separating solution (10 g washed carbon/Noritol A, Serva), 1 g dextran (Serva, molecular weight: 50,000–75,000 Da), and then 0.2 g non-fat milk powder and 100 mL of double distilled water were added to the mixture. After centrifugation at 950× for 20 min at 4 °C, the supernatants were separated, and radioactivity was determined using an NZ310 gamma counter (Gamma Ltd, Budapest, Hungary). The SOM concentrations were calculated using the standard calibration curve and expressed as fM of SOM/1 mg of total protein.

### 4.7. Statistical Analysis

For serum measurements (Luminex xMAP and ELISA), statistical significances between the control and endometriosis (stages I–IV) groups were determined using the Mann–Whitney test. The Kruskal–Wallis test followed by the Benjamini–Hochberg post hoc test was applied for all other comparisons. Statistical significance was assessed using the Mann–Whitney test for the tissue samples (Luminex xMAP, ELISA, and RIA measurements). Spearman’s rank correlation was used to assess associations between the VAS scores and the mediator concentrations. All the analyses were performed using GraphPad Prism 8.0.1, and *p*-values < 0.05 were considered statistically significant.

## 5. Conclusions

Our study using the patients’ detailed data and a subgrouping strategy provides valuable results on the systemic and local concentrations of the main endometriosis-related growth factors, cytokines, and neuropeptides and their relationships with pain severity. In conclusion systemic EGF, MIF, IL-6, and SOM might have a contribution to the development and symptomatology of endometriosis, but further rigorous and standardized research is needed to clarify their roles in disease development.

## Figures and Tables

**Table 1:** Patient demography for the serum samples.

Characteristics	CTRL (*n* = 12)	Endometriosis (*n* = 64)				
		Mild (*n* = 8)	Severe (*n* = 56)	*p*		
				Ctrl vs. Mild	Ctrl vs. Severe	Mild vs. Severe
Age	25.75 ± 4.04	31.88 ± 4.91	35.36 ± 5.95	0.0350	<0.0001	0.1327
rASRM stage						
Mild (rASRM I–II)	n.r.	8	-			
Severe (rASRM III–IV)	n.r.	-	56			
Menstrual phase						
Duration of menstrual phase (days)	29 ± 1	28	27.91 ± 0.69			
Proliferative (1–14 days)	5	4	13			
Secretory (15–28 days)	7	2	22			
Irregular	-	-	1			
No cycle	-	2	16			
No data available	-	-	4			
Parity						
SC	0	4	15			
PVN	0	-	7			
1 child	0	2	13			
2 children	0	1	4			
3 children	0	-	1			
Medication						
No oral contraceptive	12	4	36			
Progesterone	-	3	6			
Combined oral contraceptives	-	1	14			
VAS score						
DM	n.r.	7 ± 1.91	6.64 ± 3.42			0.7913
Dyspareunia	n.r.	4.14 ± 3.34	3.08 ± 3.44			0.4001
Dysuria	n.r.	2 ± 3.61	1.42 ± 2.94			0.7694
Dyschezia	n.r.	4.29 ± 4.64	2.34 ± 3.33			0.2612
CPP	n.r.	5.14 ± 3.53	2.52 ± 2.89			0.0503
No data available	n.r.	1	3			

Abbreviations: CPP: chronic pelvic pain, DM: dysmenorrhea; n.r.: non-relevant; PVN: per vias naturales; rASRM: revised American Society for Reproductive Medicine; SC: sectio caesarea.

## Data Availability

The data presented in this study are available on request from the corresponding author. The data are not publicly available due to privacy or ethical restrictions.

## Supplementary Materials

The following supporting information can be downloaded at https://www.mdpi.com/article/10.3390/ijms26146695/s1.

## Abbreviations

The following abbreviations are used in this manuscript:

CGRP	calcitonin gene-related peptide
COCs	combined oral contraceptives
CPP	chronic pelvic pain
DIER	deep-infiltrating endometriosis
DM	dysmenorrhea
EGF	epidermal growth factor
IL	interleukin
MIF	macrophage migration inhibition factor
NC	no cycle
NOCs	no oral contraceptives
PROG	progesterone
PROL	proliferative phase
PVN	per vias naturales
rASRM	revised American Society for Reproductive Medicine
SC	sectio caesarea
SEC	secretory phase
SOM	somatostatin
TNF	tumor necrosis factor
VAS	visual analogue scale
VEGF	vascular endothelial growth factor
