# Supplementary material for: Disease Severity- and Hormonal Status-Dependent Alterations of EGF and MIF in the Serum of Endometriosis Patients
Source: Int J Mol Sci. 2025 Jul 12;26(14):6695. doi: 10.3390/ijms26146695 (PMC12294931; doi:10.3390/ijms26146695)
Supplement: Supplementary file 1 [file ijms-26-06695-s001.zip › ijms-3642777-supplementary.pdf]

Supplementary material

Table of contents:

Supplementary Figure S1. Summary of the ENZIAN and rASRM scores of the patients providing the serum samples.

Supplementary Figure S2: Correlation analysis results of serum epidermal growth factor (EGF) and vascular endothelial growth factor (VEGF) in stage I-IV endometriosis.

Supplementary Figure S3: Correlation analysis results of serum macrophage migration inhibitory factor (MIF), tumor necrosis factor α (TNF-α), and interleukins (IL-1β, IL-6, IL-8) in stage I-IV endometriosis.

Supplementary Figure S4: Correlation analysis results of serum calcitonin gene-related peptide (CGRP) and somatostatin (SOM) in stage I-IV endometriosis.

Supplementary Figure S5: Correlation analysis results of serum epidermal growth factor (EGF) and vascular endothelial growth factor (VEGF) in stage I-II endometriosis.

Supplementary Figure S6: Correlation analysis results of serum macrophage migration inhibitory factor (MIF), tumor necrosis factor α (TNF-α), and interleukins (IL-1β, IL-6, IL-8) in mild (stages I-II) endometriosis.

Supplementary Figure S7: Correlation analysis results of serum calcitonin gene-related peptide (CGRP) and somatostatin (SOM) in mild (stages I-II) endometriosis.

Supplementary Figure S8: Correlation analysis results of serum epidermal growth factor (EGF) and vascular endothelial growth factor (VEGF) in severe (stages III-IV) endometriosis.

Supplementary Figure S9: Correlation analysis results of serum macrophage migration inhibitory factor (MIF), tumor necrosis factor α (TNF-α), and interleukins (IL-1β, IL-6, IL-8) in severe (stages III-IV) endometriosis.

Supplementary Figure S10: Correlation analysis results of serum calcitonin gene-related peptide (CGRP) and somatostatin (SOM) in severe (stages III-IV) endometriosis.

Supplementary Figure S11: Correlation analysis results of tissue epidermal growth factor (EGF) and vascular endothelial growth factor (VEGF) and somatostatin (SOM).

Supplementary Figure S12: Correlation analysis results of tissue macrophage migration inhibitory factor (MIF), tumor necrosis factor α (TNF-α), and interleukins (IL-1β, IL-6, IL-8).

Supplementary Table S1: The intra- and inter-assay variation coefficients of the Milliplex Human Cytokine/Chemokine Magnetic Bead Panel multiplex assay.

Supplementary Table S2: The intra- and inter-assay variation coefficients of the CGRP, MIF, and SOM ELISA assays.

Supplementary Figure S1. Summary of the ENZIAN and rASRM scores of the patients providing the serum samples.

A

Patient ID	ENZIAN	rASRM	
		Stage	Points
M1	P1	I	4
M2	P1	I	4
M3	P1	I	4
M4	P1 C1	II	12
M5	P1B1	II	10
M6	A1FA	II	9
M7	A1	II	10
M8	A2	II	10
S1	A3C2	III	28
S2	C1FA	III	28
S3	P1A1	III	29
S4	A1C2	III	18
S5	A1C2	III	22
S6	P3O1A2FAFI	III	32
S7	A2C3FAFI	III	24
S8	O2B1	III	26
S9	A1C2	III	42
S10	A1FAFBFI	III	28
S11	A1B2FA	III	27
S12	P1A1C2	III	33
S13	A1C3	III	31
S14	P2O	III	22
S15	O2B1	III	18
S16	A2C2FA	III	29
S17	A1FA	III	18
S18	A3C3FI	III	34
S19	A1C1FA	III	32
S20	P1O2FI	IV	40
S21	A2C1FA	III	30
S22	C3FA	III	22
S23	FAFI	IV	40
S24	A2C2FO	III	29
S25	A3C2FA	III	16
S26	A1C2FAFI	IV	49
S27	A1C2FA	IV	41
S28	A3C2FAFBFI	IV	67
S29	A1C2FI	IV	65
S30	A2C3FA	IV	82
S31	A2C2FUFA	IV	67
S32	A1C2	IV	56
S33	A3B2C3FIFUFA	IV	69
S34	A2C2FA	IV	69
S35	A3C3FAFB	IV	41
S36	A2B2C3FA	IV	43
S37	A1C2FIFAFB	IV	76
S38	A2C2FA	IV	49
S39	A3B2C3FAFI	IV	110
S40	A2C2FAFBFI	IV	49
S41	A3C3FA	IV	49
S42	P2O2/2A2	IV	46
S43	A1C3	IV	46
S44	A1C3	IV	46
S45	A1FI	IV	43
S46	A3B3C3FA	IV	66
S47	A3C3FAFIFU	IV	53
S48	A2C1FAFUFB	IV	47
S49	A1C2FBFA	IV	42
S50	A1C2FA	IV	66
S51	A2C3FAFB	IV	47
S52	A3B3C3FAFUFI	IV	66
S53	P2O2/0A2	IV	44
S54	P2A2C2FIFB	IV	51
S55	A3B2C3FI	IV	62
S56	P1O2/2FI	IV	60

B

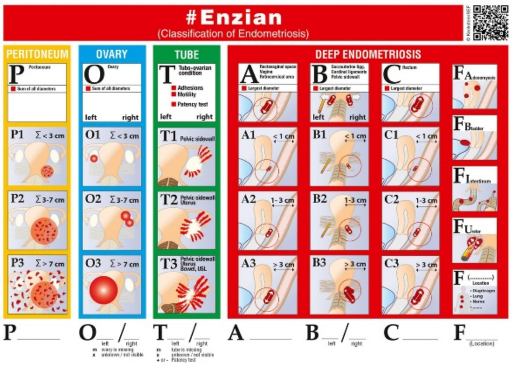

A: Table summarizing the individual ENZIAN and revised American Society for Reproductive Medicine (rASRM) scores of the patients from whom serum samples were obtained.

B: Schematic illustration of the ENZIAN scoring system. The figure was adapted from Keckstein et al.: The #Enzian classification: A comprehensive non-invasive and surgical description system for endometriosis *[5].*

Supplementary Figure S2: Correlation analysis results of serum epidermal growth factor (EGF) and vascular endothelial growth factor (VEGF) in stage I-IV endometriosis.

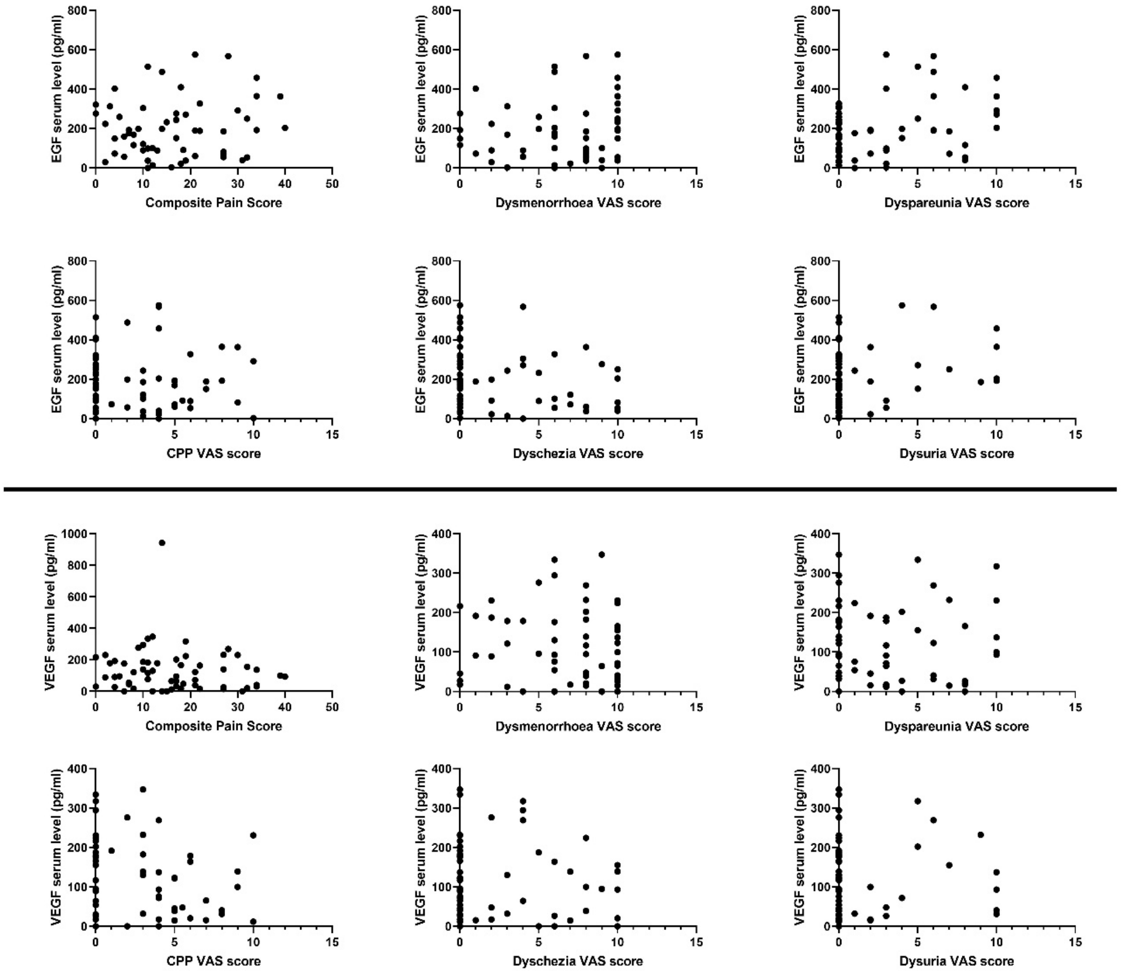

Supplementary Figure S3: Correlation analysis results of serum macrophage migration inhibitory factor (MIF), tumor necrosis factor α (TNF-α), and interleukins (IL-1β, IL-6, IL-8) in stage I-IV endometriosis.

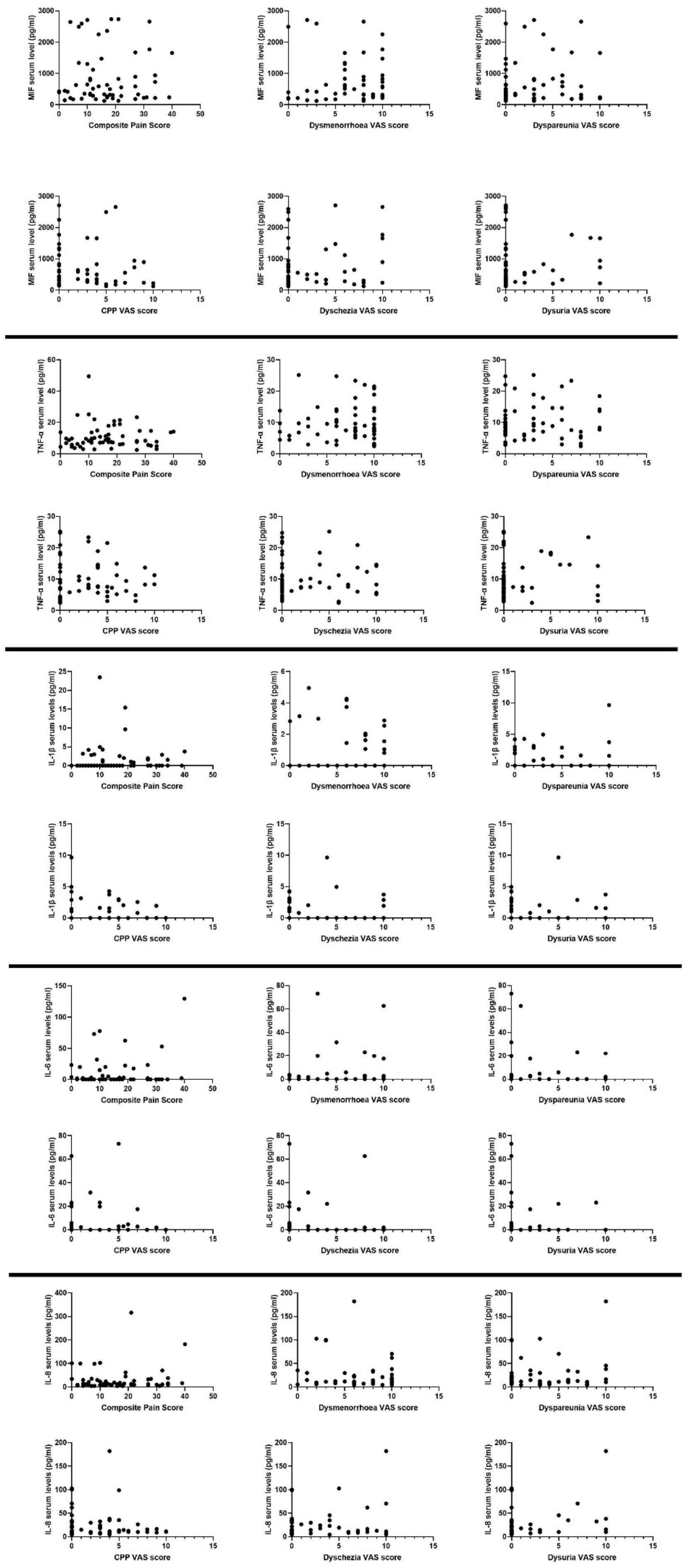

Supplementary Figure S4: Correlation analysis results of serum calcitonin gene-related peptide (CGRP) and somatostatin (SOM) in stage I-IV endometriosis.

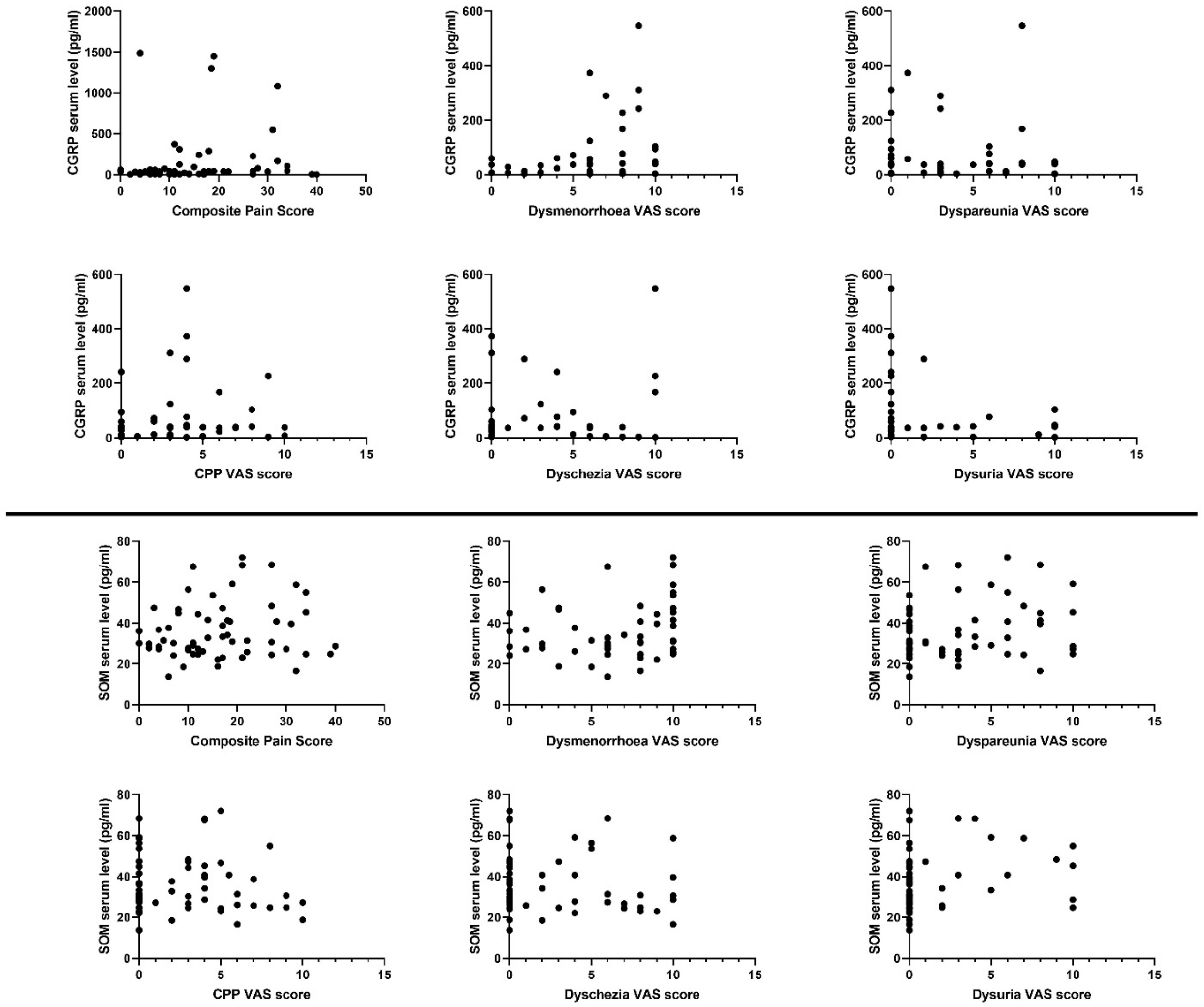

Supplementary Figure S5: Correlation analysis results of serum epidermal growth factor (EGF) and vascular endothelial growth factor (VEGF) in stage I-II endometriosis.

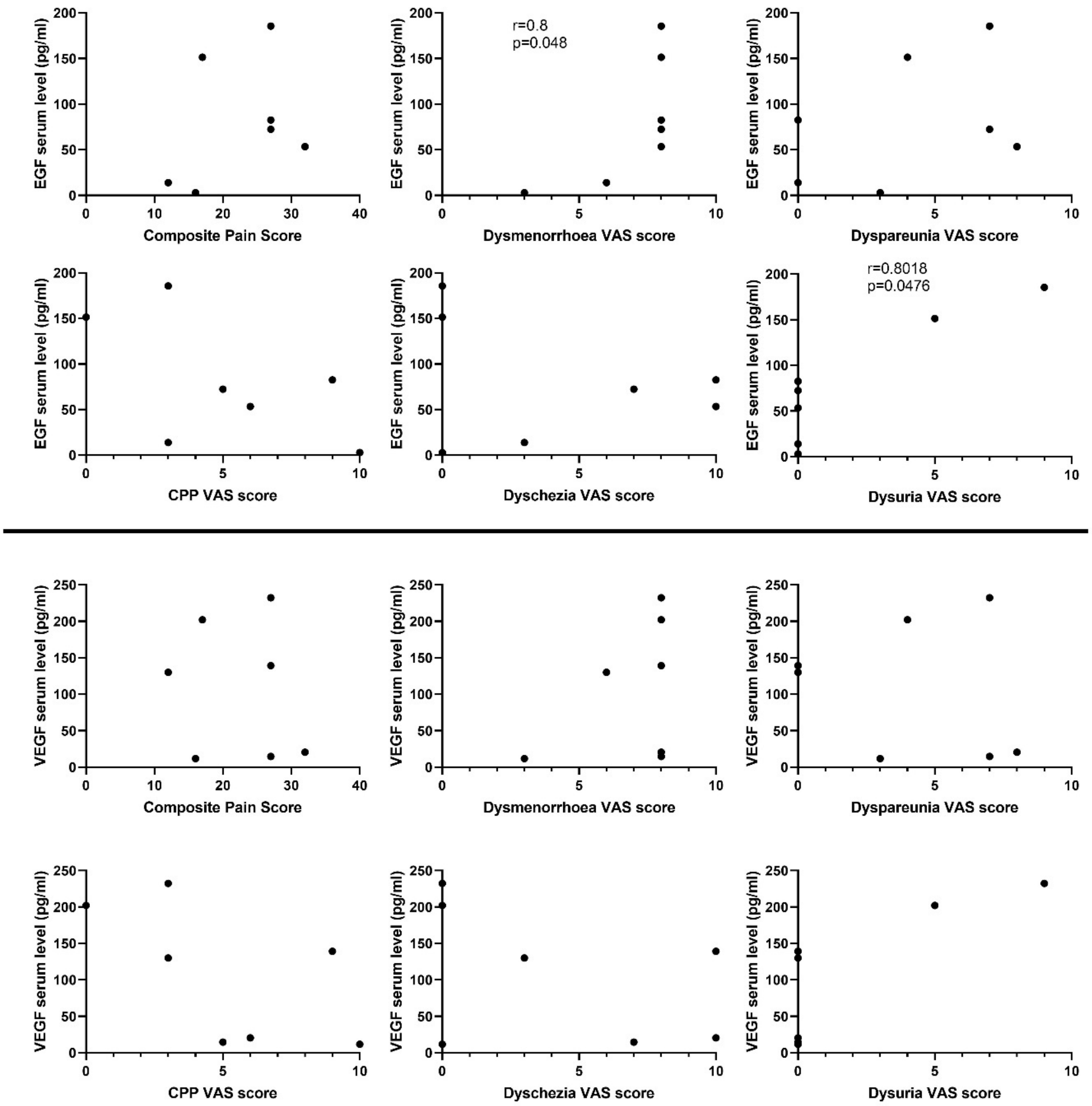

Supplementary Figure S6: Correlation analysis results of serum macrophage migration inhibitory factor (MIF), tumor necrosis factor α (TNF-α), and interleukins (IL-1β, IL-6, IL-8) in mild (stages I-II) endometriosis.

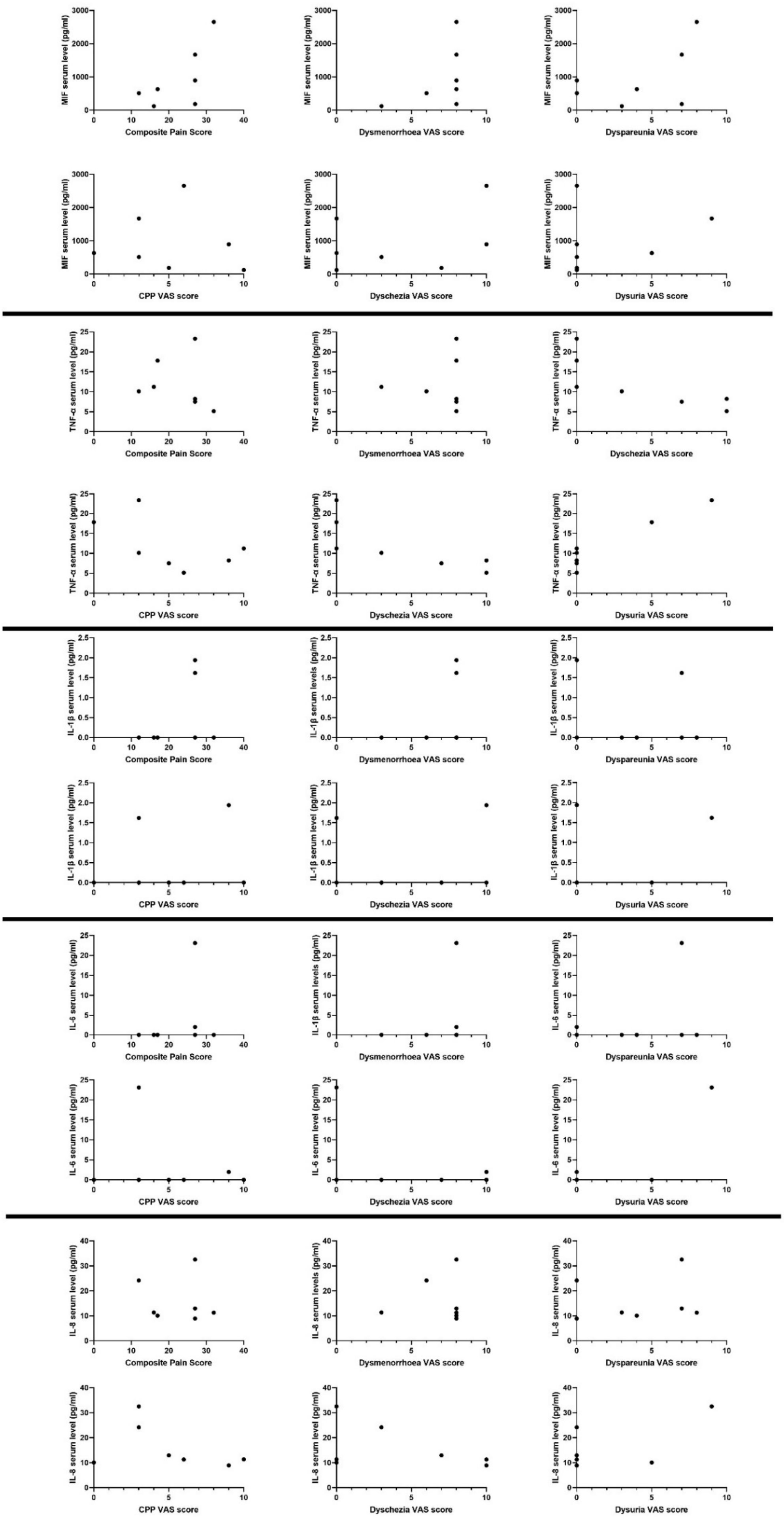

Supplementary Figure S7: Correlation analysis results of serum calcitonin gene-related peptide (CGRP) and somatostatin (SOM) in mild (stages I-II) endometriosis.

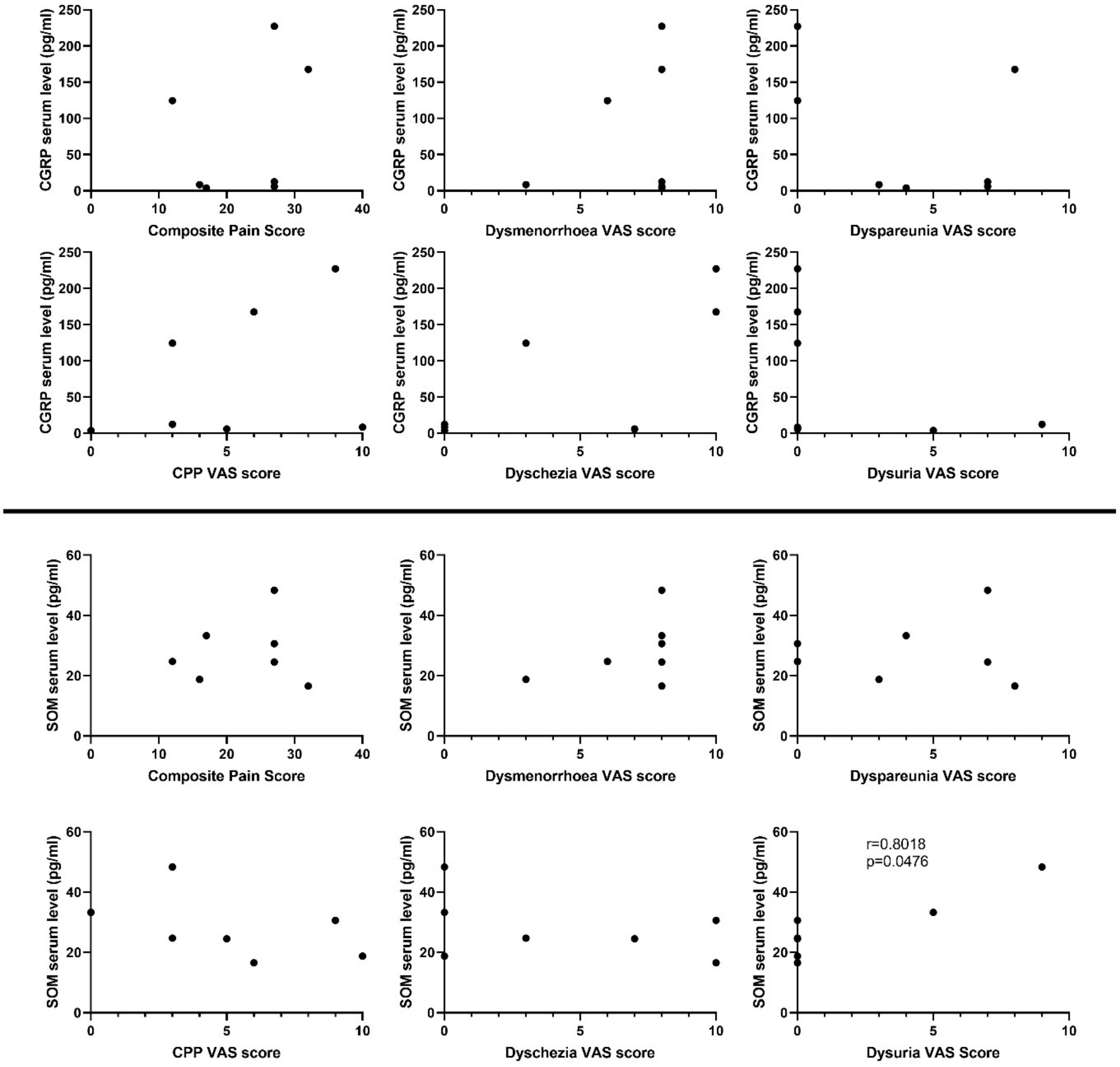

Supplementary Figure S8: Correlation analysis results of serum epidermal growth factor (EGF) and vascular endothelial growth factor (VEGF) in severe (stages III-IV) endometriosis.

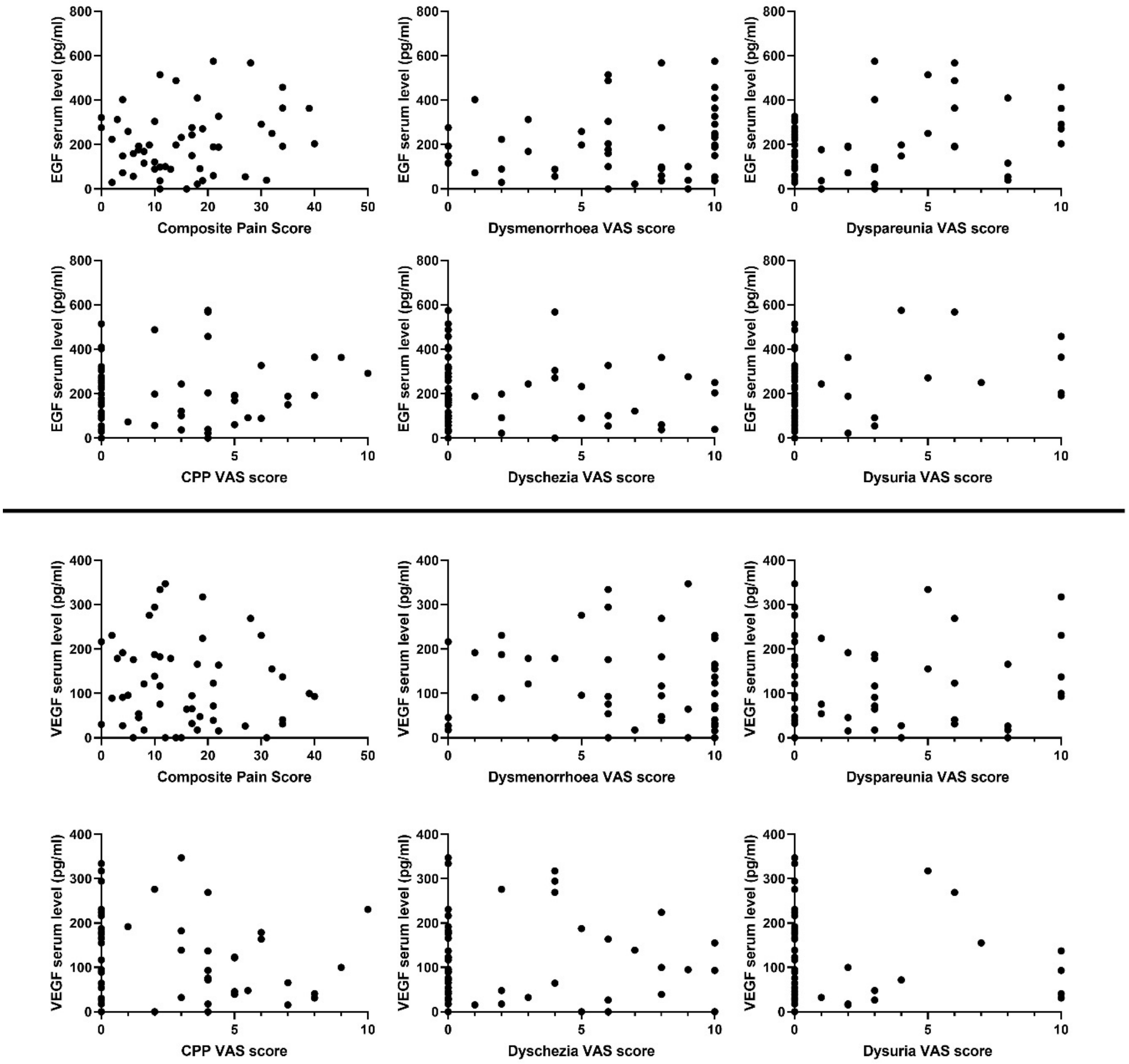

Supplementary Figure S9: Correlation analysis results of serum macrophage migration inhibitory factor (MIF), tumor necrosis factor α (TNF-α), and interleukins (IL-1β, IL-6, IL-8) in severe (stages III-IV) endometriosis.

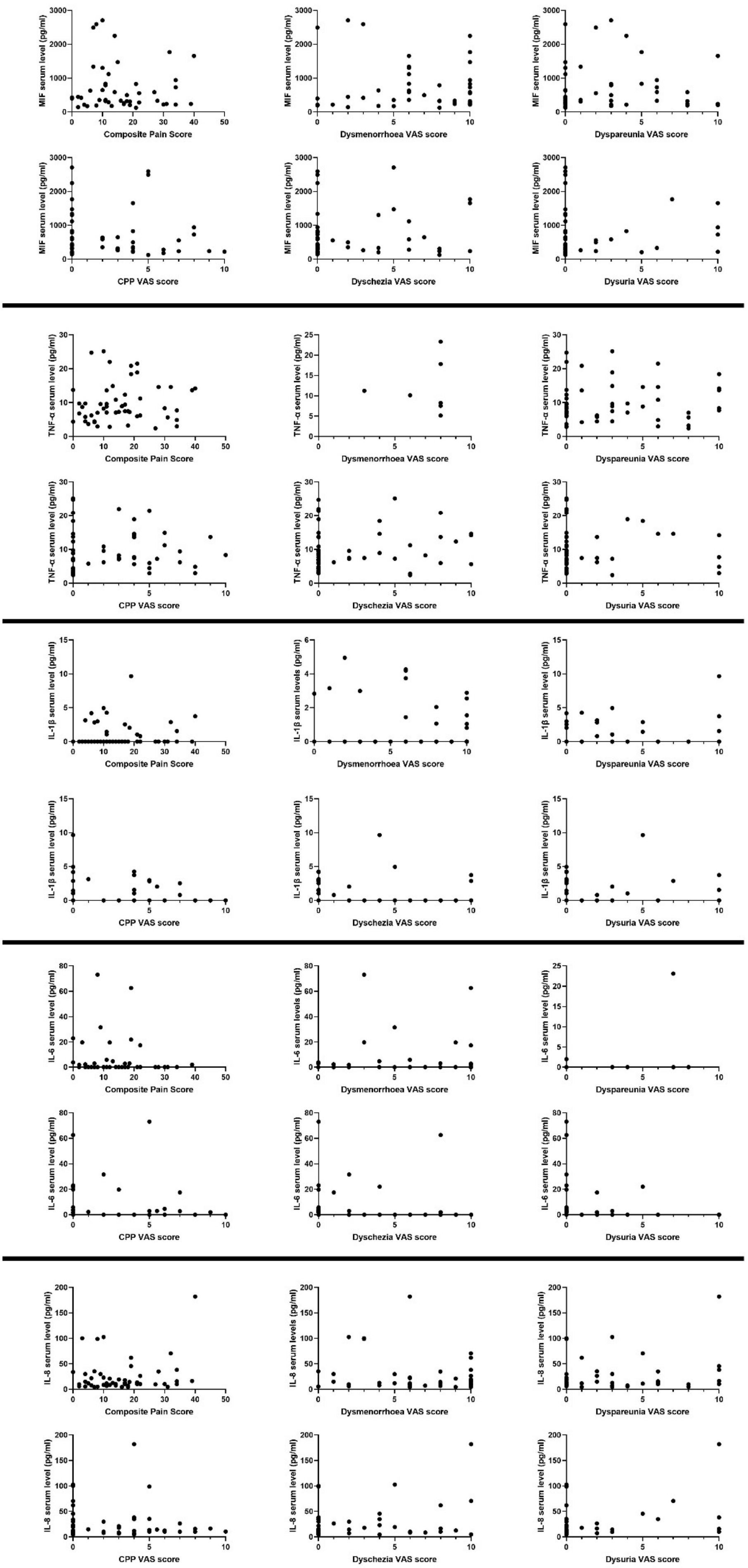

Supplementary Figure S10: Correlation analysis results of serum calcitonin gene-related peptide (CGRP) and somatostatin (SOM) in severe (stages III-IV) endometriosis.

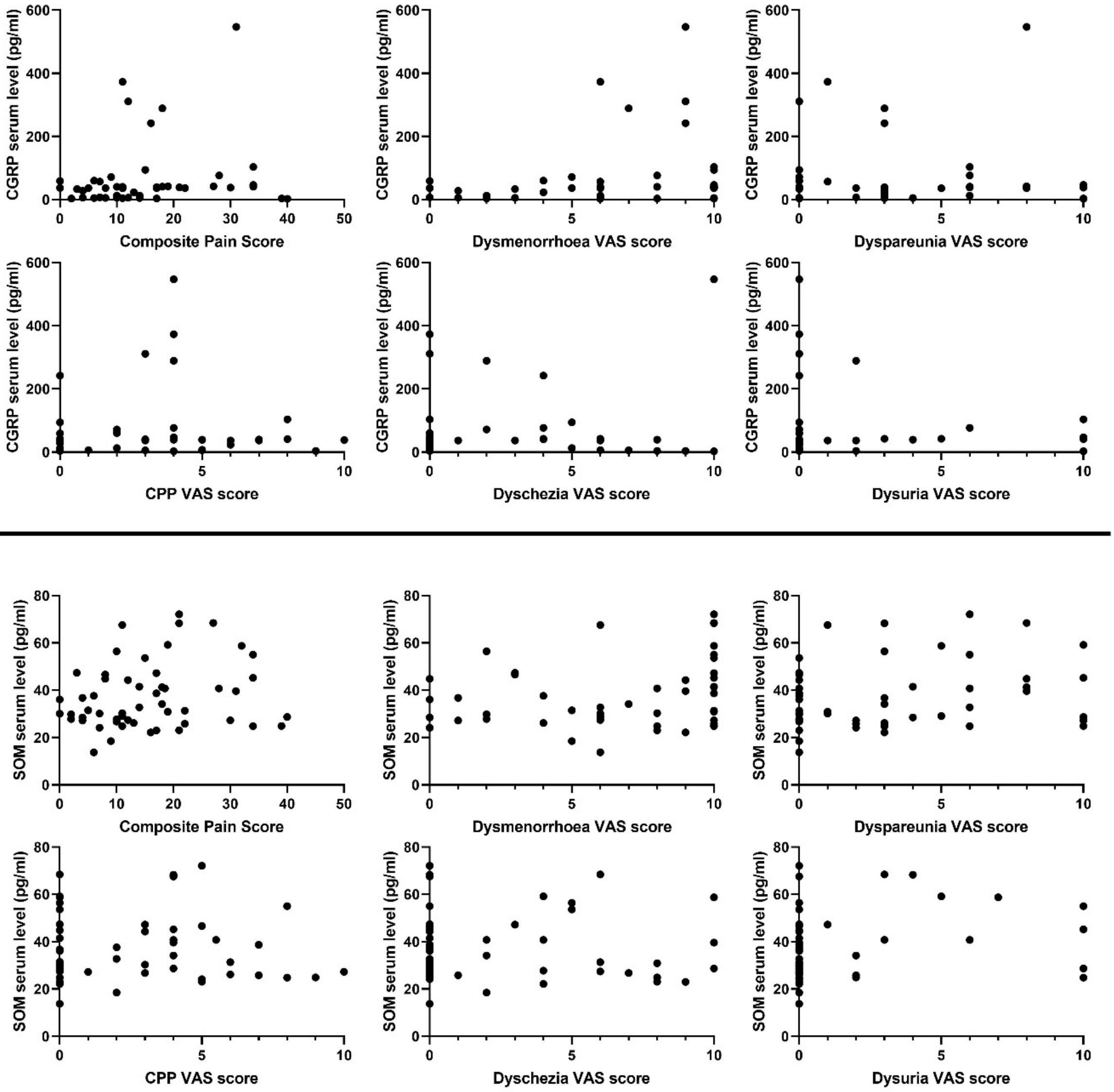

Supplementary Figure S11: Correlation analysis results of tissue epidermal growth factor (EGF) and vascular endothelial growth factor (VEGF) and somatostatin (SOM).

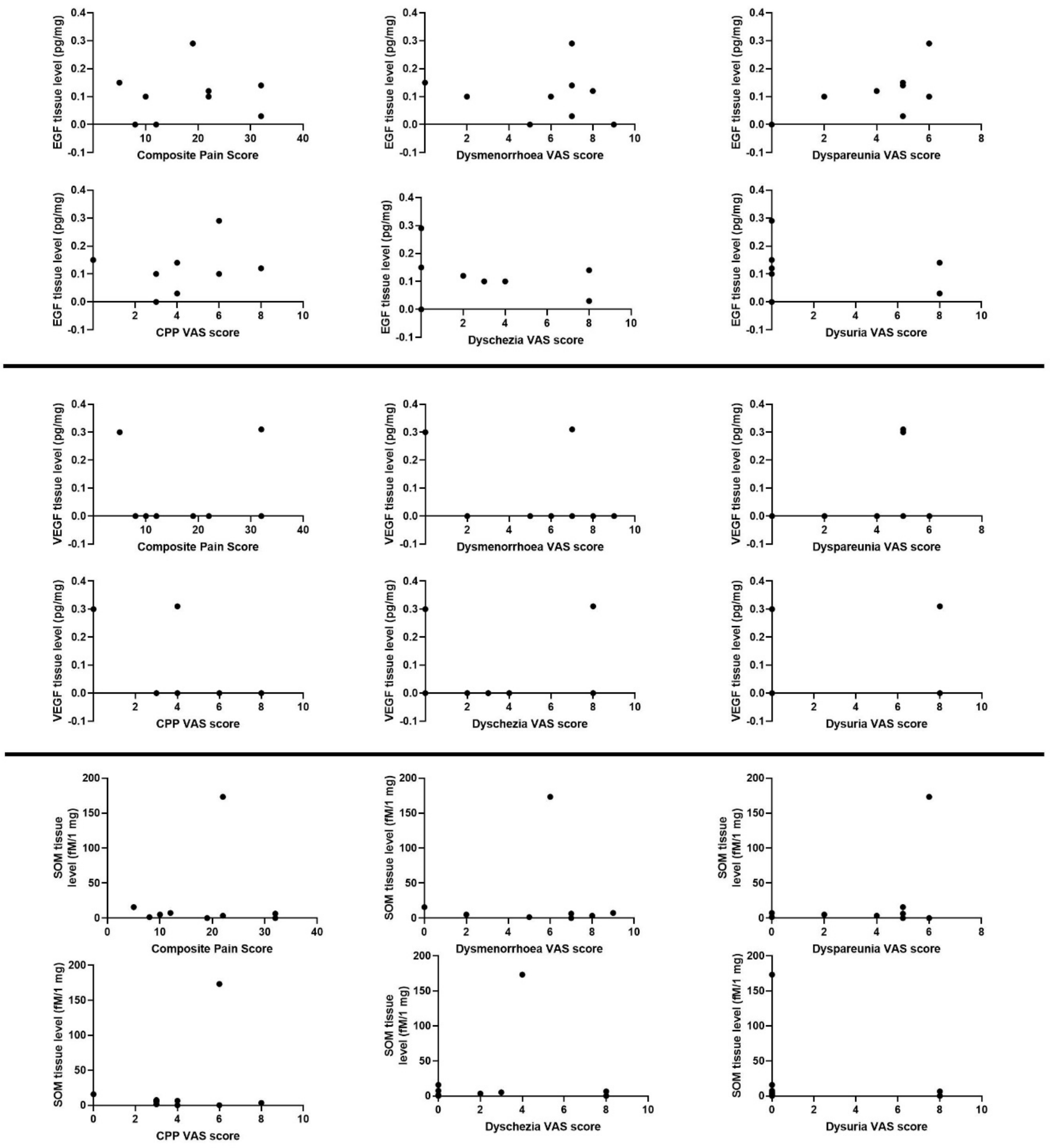

Supplementary Figure S12: Correlation analysis results of tissue macrophage migration inhibitory factor (MIF), tumor necrosis factor α (TNF-α), and interleukins (IL-1β, IL-6, IL-8).

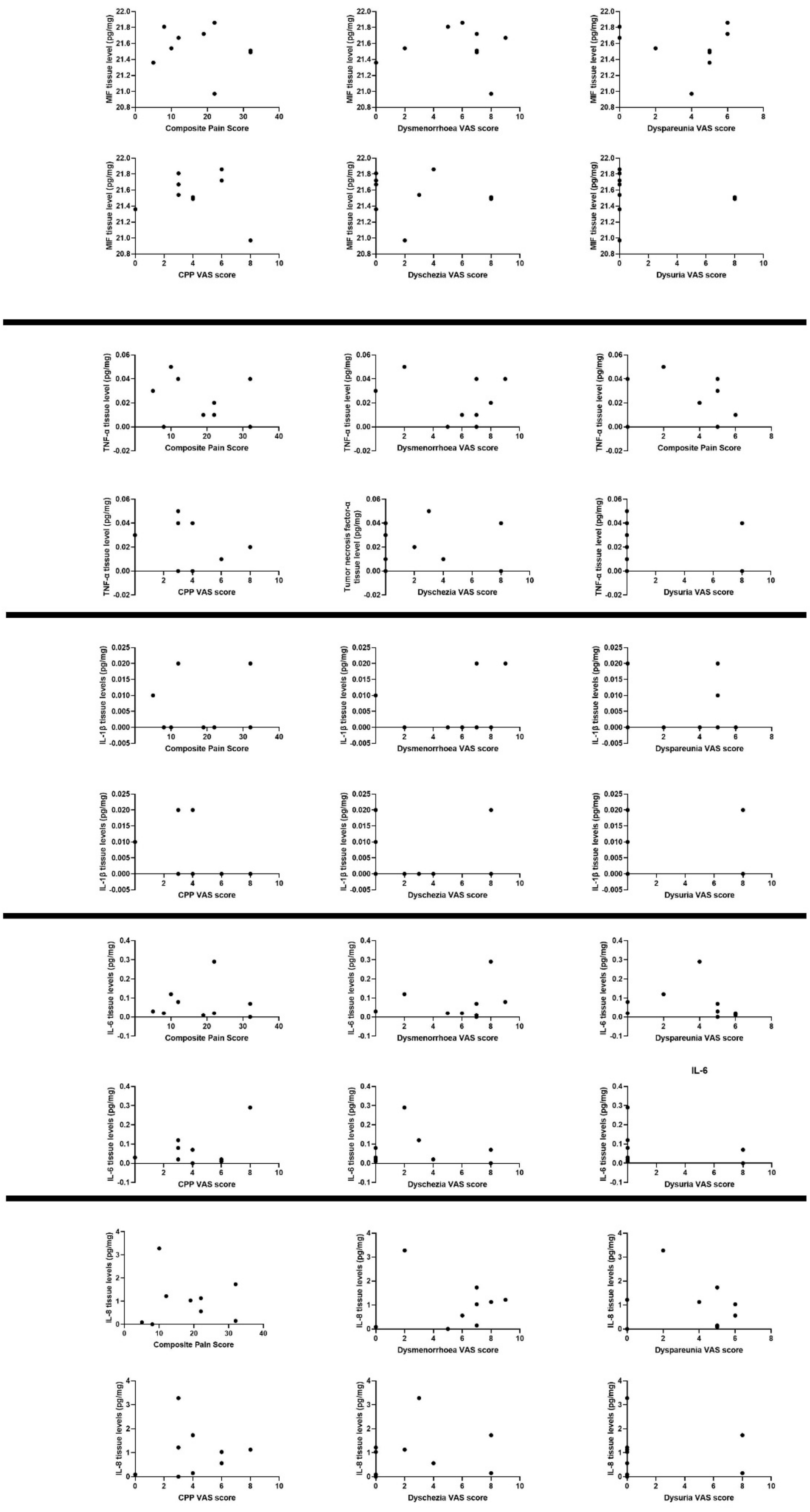

Supplementary Table S1: The intra- and inter-assay variation coefficients of the Milliplex Human Cytokine/Chemokine Magnetic Bead Panel multiplex assay.

Cytokine	Intra-assay CV	Inter-assay CV (N=6 assays)
EGF	2.3%	5.8%
VEGF	3.7%	10.4%
IL-1β	2.3%	6.7%
IL-6	2.0%	18.3%
IL-8	1.9%	3.5%
TNFα	2.6%	13.0%

Supplementary Table S2: The intra- and inter-assay variation coefficients of the CGRP, MIF, and SOM ELISA assays.

Cytokine	Intra-assay CV	Inter-assay CV
CGRP		
7.81 pg/ml	>25%	16.6%
15.6 pg/ml	20.6%	12.2%
31.3 pg/ml	7.0%	6.3%
62.5 pg/ml	5.2%	4.3%
125 pg/ml	3.3%	2.8%
250 pg/ml	3.0%	0.7%
500 pg/ml	2.7%	4.1%
1000 pg/ml	3.4%	-
MIF	<10%	<12%
SOM	<10%	<12%
